# Anterior Gradient Protein-2 Is a Regulator of Cellular Adhesion in Prostate Cancer

**DOI:** 10.1371/journal.pone.0089940

**Published:** 2014-02-27

**Authors:** Diptiman Chanda, Joo Hyoung Lee, Anandi Sawant, Jonathan A. Hensel, Tatyana Isayeva, Stephanie D. Reilly, Gene P. Siegal, Claire Smith, William Grizzle, Raj Singh, Selvarangan Ponnazhagan

**Affiliations:** 1 Department of Pathology, University of Alabama at Birmingham, Birmingham, Alabama, United States of America; 2 Hospital Laboratories, University of Alabama Hospital, Birmingham, Alabama, United States of America; 3 Vivo Biosciences Inc., Birmingham, Alabama, United States of America; Thomas Jefferson University, United States of America

## Abstract

Anterior Gradient Protein (AGR-2) is reported to be over-expressed in many epithelial cancers and promotes metastasis. A clear-cut mechanism for its observed function(s) has not been previously identified. We found significant upregulation of AGR-2 expression in a bone metastatic prostate cancer cell line, PC3, following culturing in bone marrow-conditioned medium. Substantial AGR-2 expression was also confirmed in prostate cancer tissue specimens in patients with bone lesions. By developing stable clones of PC3 cells with varying levels of AGR-2 expression, we identified that abrogation of AGR-2 significantly reduced cellular attachment to fibronectin, collagen I, collagen IV, laminin I and fibrinogen. Loss of cellular adhesion was associated with sharp decrease in the expression of α4, α5, αV, β3 and β4 integrins. Failure to undergo apoptosis following detachment is a hallmark of epithelial cancer metastasis. The AGR-2-silenced PC3 cells showed higher resistance to Tumor necrosis factor-related apoptosis- inducing ligand (TRAIL) induced apoptosis *in vitro*. This observation was also supported by significantly reduced Caspase-3 expression in AGR-2-silenced PC3 cells, which is a key effector of both extrinsic and intrinsic death signaling pathways. These data suggest that AGR-2 influence prostate cancer metastasis by regulation of cellular adhesion and apoptosis.

## Introduction

Prostate cancer has the highest incidence rate among all cancers in men in the developed world and is the third leading cause of death behind lung and colorectal cancers [1]. Pathogenesis of prostate cancer and its preferential metastasis to bone and other organs are regulated by genes, which function cooperatively to promote each step of carcinogenesis and the metastatic cascade [2]–[4]. Although distinct gene signatures governing stages of cancer progression are reported in breast cancer, such information regarding prostate cancer is still lacking [5]. Identifying the role of new molecules of diagnostic and therapeutic significance remains a major focus of current cancer research. By gene expression analysis, we observed significant augmentation of AGR-2 in the PC3 prostate cancer cell line, metastasized to bone, following maintenance in normal bone marrow-conditioned medium. AGR-2 was first identified as XAG-2 in *Xenopus laevis*, which induces differentiation of cement gland that helps the embryos to remain attached to substratum through secretion of a mucinous substance [6]. The mammalian homologue, AGR-2, is a protein disulfide isomerase (PDI), which contains a thio-redoxin domain and responsible for intestinal mucus production [7]. Mice lacking AGR-2 are prone to develop colitis [7]. AGR-2 has drawn considerable attention in recent years for its putative role in neoplastic progression and metastasis [8]–[15]. AGR-2 has been found to be over-expressed in various adenocarcinomas and has been linked to poor survival of patients with breast and prostate cancers [16], [17]. Few recent reports have shed light on the regulatory elements of AGR-2 function, but its specific role(s) in tumorigenesis and progression to metastasis is still lacking [18]–[20]. In prostate cancer, AGR-2 is reported to be androgen inducible and only over-expressed during early stages of carcinogenesis [13], [21]. On the contrary, a recent study also observed reduced AGR-2 expression in high grade tumors, which coincided with metastatic disease [13].

The present study employed AGR-2 gene silencing method in bone metastatic human prostate cancer cell line PC3 to understand its biological function in prostate cancer bone metastasis. Results indicated loss of AGR-2 in PC3 cells resulted in significant reduction in tumor cell adhesion, loss of expression of α4, α5, αV, β3 and β4 integrins and development of apoptosis resistance, suggesting role of AGR-2 in metastatic cascade of prostate cancer by affecting tumor cell adhesion and migration.

## Materials and Methods

### Cell Lines and Reagents

An osteolytic human prostate cancer cell line, PC3, was purchased from ATCC (Manassas, VA), and a clonal derivative of the PC3 cells, expressing firefly luciferase, was a generous gift from Dr. Kenneth J. Pienta (University of Michigan, Ann Arbor, Michigan). Both cell lines were maintained in RPMI-1640 medium (Mediatech Inc. Hendron, VA) supplemented with 10% fetal bovine serum (Mediatech Inc.) and penicillin/streptomycin (Mediatech Inc). Total RNA was isolated using Trizol (Invitrogen, Carlsbad, CA) and purified using a QIAGEN mini kit (Valencia, CA). cDNA microarray analysis was performed by the Cancer Genomics Shared Resources at the Winship Cancer Center of Emory University (Atlanta, GA) using an Illumina Beadstation 500 and data were analyzed by JAVA Treeview, Spotfire, Gene Cluster 3.0 and Ingenuity pathway analysis. iScript cDNA synthesis kit was purchased from Bio-Rad (Hercules, CA). AGR-2 primers (Forward 5′-TTGGGGTGACCAACTCATCT-3′; Reverse 5′- GGACAAACTGCTCTGCCAAT-3′) for RT-PCR analysis were designed using the Primer 3 (version 4.0) software and oligonucleotides were purchased from Integrated DNA Technologies, Inc. (Coralville, IA). cDNA samples were analyzed in Bio-Rad iCycler (Hercules, CA). 3-dimensional (3-D) PC3 beads for growth in bone marrow conditioned medium were generated following growth of PC3 cells on hu-biogel matrices and supplied by Vivo Biosciences (Birmingham, AL). A Vybrant ® MTT Cell Proliferation Assay Kit was purchased from Molecular Probes, Inc. (Eugene, OR). 3-D Culture Matrix™ basement membrane extract was purchased from Cultrex (3445-00501, Gaithersburg, MD). A Cytoselect™ 48-well cell adhesion assay kit was purchased from Cell Biolabs, Inc. (CBA-070, San Diego, CA). All kits and reagents were used following manufacturer’s instructions.

### Antibodies

Mouse and human monoclonal antibodies, and polyclonal antibodies for AGR-2 were purchased from Abcam Ltd (Cambridge, MA, and used at a dilution of 1∶1000 for immunoblots and 1∶500 for IHC). An integrin antibody sampler kit was purchased from Cell Signaling (4749S, Danvers, MA, and used at a dilution of 1∶1000 for immunoblots and 1∶500 for IHC). Caspase-3, Cleaved Caspase-3 and beta-actin antibodies were purchased from Cell Signaling (Danvers, MA, and used at a dilution of 1∶500 for immunoblots). Secondary immuno-detection was performed using goat-anti-rat/mouse/rabbit IgG ABC kits purchased from Vector Laboratories (Burlingame, CA) and GE Healthcare (Buckinghamshire, UK). For immunofluorescence secondary detection, goat-anti-rat/mouse/rabbit IgG labeled with Alexa-Fluor-594 was purchased from Molecular Probes, Inc. (Eugene, OR, 2 µg/ml).

### Human Tissue Samples

Bone metastatic prostate cancer tissue was obtained from autopsy at University of Alabama at Birmingham in accordance with approved institutional review board (IRB) protocol. Formalin-fixed paraffin embedded tissue blocks obtained from these sources were sectioned at 6 µm. All tissue samples were reviewed and characterized by board-certified anatomic pathologists.

### Isolation of Bone Marrow-conditioned Medium

Male C57BL/6 mice were sacrificed and both femur and tibiae were harvested and marrow was flushed out with sterile serum-free SIGMA Stemline medium (St Louis, MO) using a 28.5 gauge needle fitted to a 1 ml syringe in a 50 ml sterile tube containing 10 ml of serum-free Stemline medium. Bone marrow clumps were removed by repeated passing through a 16.5 gauge needle fitted to a 10 ml syringe until a homogeneous single cell suspension is achieved. The cells are pelleted via centrifugation at 1200 rpm, washed three times using serum-free medium and finally re-suspended in 50 ml Stemline medium containing 10% fetal bovine serum and antibiotics. The cells are further diluted with serum containing Stemline medium to 100 ml and distributed to four 150 cm^2^ cell-culture flasks (Corning Inc. Corning, NY) for maintenance in a moist chamber with 95% O_2_ and 5% CO_2_ supply. After two days the culture medium was collected and centrifuged at high speed to get rid of cells and debris. The supernatants were pooled and used directly for the culture of 3-D, PC3 beads.

### Construction of Recombinant shRNA Vector and Development of Single Cell-derived PC3 Clones with AGR-2 Knockdown

Twenty one-base pair targets were selected from unique regions of the AGR-2 coding sequence using Tuschl et al., 1999 siRNA design guidelines [22]. Among the tested shRNA sequences, the one that showed maximum efficacy was used in the present study and consisted of 21-mer sense (GACAAACACCTTTCTCCTGAT) and anti-sense strands separated by a 9-bp region and contained BamHI or Hind III restriction sites, respectively, for directional cloning (Integrated DNA Technologies, Coralville, IA). The oligonucleotides were annealed and directly ligated into a pRNAT.U6/Neo/GFP expression plasmid (Genescript, Piscataway, NJ). One of the 21-mer sequences (CCTTGAGACTTGAAACCAGAA) that failed to silence AGR-2 expression was used as the experimental control to minimize any off-target effect. PC3 cells were cultured in 6-well plates to approximately 90% confluence in antibiotic free medium. The cells were then transfected with AGR-2 shRNA expressing plasmids using Lipofectamine 2000 (Invitrogen, Carlsbad, CA) according to the manufacturer’s instructions. After 24 hr, the transfection medium was replaced with complete medium and after another 24 hrs, GFP positive cells were flow sorted and maintained in pre-standardized 600 µg/ml neomycin (10131, GIBCO, Invitrogen, Carlsbad, CA). Once neomycin resistant cells developed, cells were trypsinized and again plated into 96-well culture dishes at a 1 cell/well frequency, in duplicates. AGR-2 knockdown PC3 clones (PC3^AGR−2sh^) and control cell clones (PC3^Control^) were selected after testing for AGR-2 expression via western blotting and immunofluorescence analyses.

### sTRAIL-induced Apoptosis Assays

Recombinant human TNF-related apoptosis-inducing ligand (TRAIL) was purchased from EMD Millipore Corporation, Temecula, CA. PC3^Control^ and PC3^AGR−2sh^ cells were treated with 0, 12.5, 25, 50 and 100 ng/ml concentration of TRAIL. Cell viability was determined after 12 hrs and 72 hrs by propidium iodide staining (BD Biosciences) and MTT Cell Proliferation Assay Kit respectively. Treated cells were also harvested after 0 hr, 1 hr, 3 hrs and 6 hrs by cell scraping, cell pellets were washed twice with ice-cold PBS and subjected to Western blotting for the detection of caspase 3 and active cleaved caspase-3 peptides.

### Western Blotting

Cells were trypsinized, washed and re-suspended in protein lysis buffer prior to being freeze-thawed once at −80°C. Proteins were denatured adding SDS-PAGE buffer containing β-mercaptoethanol and incubating at 95°C for 5 min. Genomic DNA was sheared by ultra-sonication. Protein concentrations were measured using Lowry’s method (Biorad, Hercules, CA). Proteins were separated in either 10 or 15% polyacrylamide gel and transferred to nitrocellulose membrane. The membrane was incubated for 1 hr, in 2% non-fat dried milk, in TBST to block non-specific primary antibody binding. The membrane was then incubated overnight with appropriately diluted primary antibodies in TBST buffer. Beta-actin antibody was used as the loading control. Following a 3 times wash in TBST, the membrane was incubated in goat anti-mouse/rabbit IgG conjugated to horse radish peroxidase (1∶5000, Agilent Technologies, Santa Clara, CA) for 30 min. The membrane was washed again in TBST and developed using an enhanced chemiluminescence reagent (GE Healthcare Bio-Sciences Corp. Piscataway, NJ) and imaged on a Fuji LAS-3000 chemiluminescence developer.

### Immunofluorescence

Both PC3^control^ and PC3^AGR−2sh^ cells were grown in culture until 50% confluence in chambered slides, washed thoroughly in PBS and fixed in ice-cold 3.7% paraformaldehyde in PBS containing 0.1% Triton-X-100, for 20 min, at room-temperature. Cells were washed thoroughly and incubated overnight at 4°C in rat monoclonal human AGR-2 antibody. Following PBS wash, cells were incubated with alexa-fluor-594 labeled anti-rat IgG (Molecular Probes, Eugene, OR), for 30 minutes, at room temperature. Cells were washed and nuclei were stained with DAPI for contrast. The fluorescent labeled cells were mounted using Vectashield (Vector Labs, Burlingame, CA) and viewed in a Leica DMRB fluorescence microscope.

### Histomorphometry and Immunohistochemistry

Soft tissues were fixed in 10% neutral buffered-formalin solution for 48 hours before embedding in paraffin for histological analysis. Bone tissues were decalcified in 0.5 mol/L EDTA in Ca^2+^- and Mg^2+^-free Dulbecco’s PBS (Cellgro) prior to embedding in paraffin. Six µm longitudinal serial sections were cut from the femur and tibia and stained with hematoxylin and eosin (H&E) to determine the characteristics of tumor growth in the bone. For immunohistochemistry, 6 µM paraffin sections were deparaffinized in xylene, and hydrated through graded-alcohol. Antigen retrieval was performed in citrate buffer, pH 6.0, under steam for 20 min. Sections were cooled to room temperature and endogenous peroxidase was removed using 0.3% H_2_O_2_ in methanol for 30 min and blocked with 3% normal goat serum for 30 min. Tissue sections were then incubated with primary antibodies overnight at 4°C. Sections were washed in PBST and again incubated at room temperature (RT) with biotin-conjugated goat anti-rabbit/anti-rat secondary antibody for 2 hrs. After washing, sections were incubated with streptavidin-conjugated horseradish peroxidase for 1 hr at room temperature. After another wash with PBST, immunodetection was performed using DAB-H_2_O_2_ (Vector Labs, Burlingame, CA) and counterstained with hematoxylin wherever applicable.

### Migration Assays

Migration assays were performed by a “wound closure” method and by using Boyden chamber assay. In the wound closure assay, AGR-2-silenced PC3 cells and control PC3 cells were plated at 10^5^ per well in a six-well plate. Upon reaching 90% confluency, the cells were serum starved overnight. Fresh medium was added to the cells and wounds were created using a sterile 200 µl pipette tip. Photographs of the wound closure were taken at 0 hrs and 22 hrs to compare the difference in the rate of wound closure between the AGR-2-silenced and control PC3 cell lines. A Boyden chamber migration assay was performed by plating 4×10^4^ PC3^control^ and PC3^AGR−2sh^ cells per well on a cell culture insert (8 µm pore size, 24-well format; Becton Dickinson Labware) in serum-free medium in triplicate. To initiate migration 10% FBS was used as a chemo-attractant in the lower chamber. Cells were incubated for 6 hrs at 37°C and removed from the upper chamber using a cotton swab. Cells on the underside of the chamber were visualized under a fluorescence microscope and counted using Image J software. The effect of AGR-2 silencing on PC3 cell migration was presented as relative values compared to control (100%).

### Statistical Analysis

Data were analyzed by Student’s *t*-test. Values provided are the Mean ± SEM and the differences were considered significant if p<0.05.

## Results

### AGR-2 Expression is Increased in Metastatic Prostate Cancer Cells in the Presence of Conditioned Bone Marrow Microenvironment

**Figure 1:**
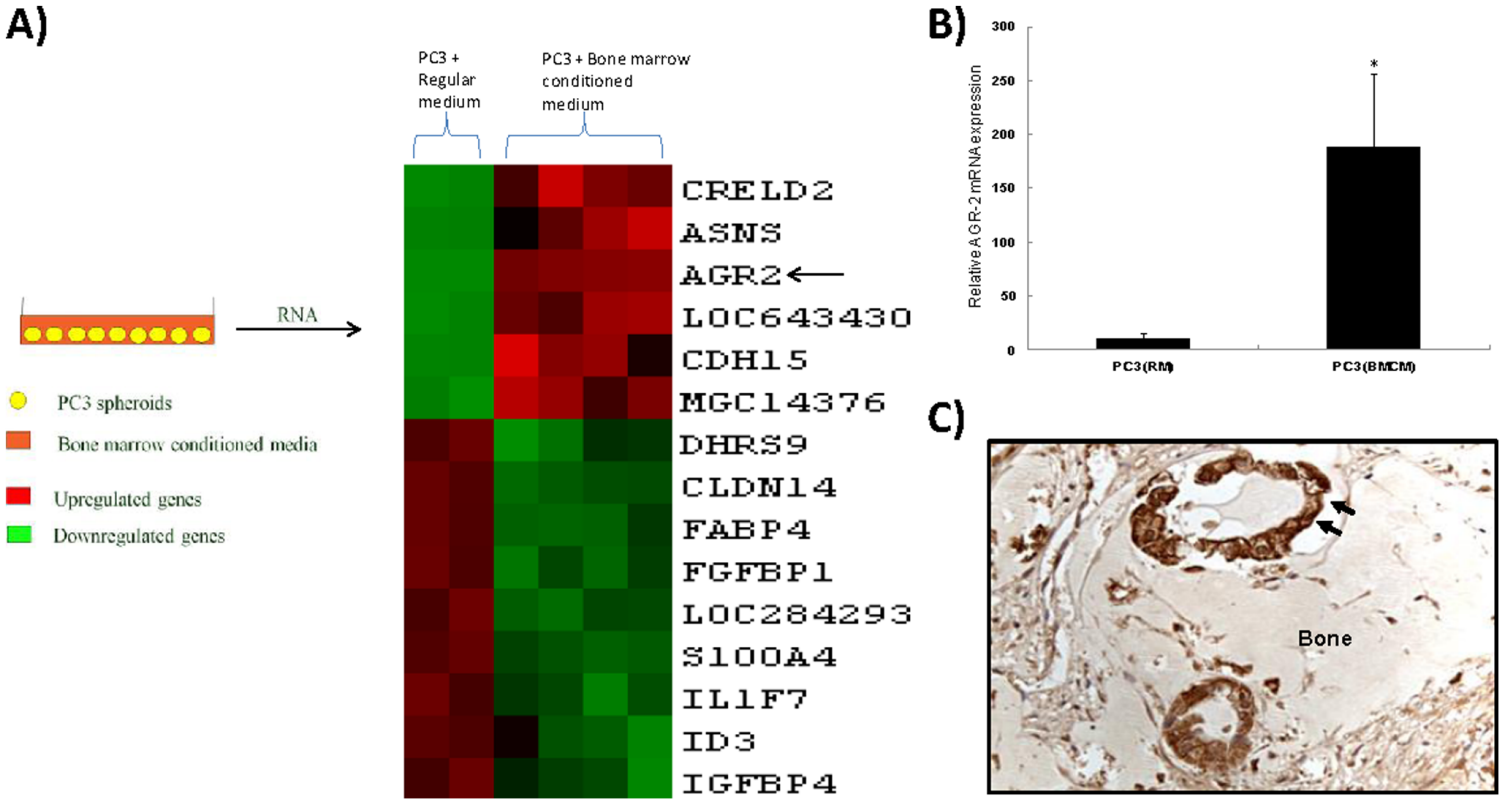
AGR-2 over-expression in PC3 cells following growth in bone marrow conditioned medium. **A**. cDNA microarray heat map showing higher AGR-2 expression (red) in PC3 cells grown in bone marrow conditioned medium compared to regular medium (green). **B**. Real-time RT-PCR data showing a significant (p<0.05) increase in AGR-2 expression in PC3 cells following growth in normal bone marrow conditioned medium (BMCM) as compared to when grown in regular medium (RM). **C**. Immunohistochemical analysis showing intense AGR-2 immunostaining in the human prostate cancer cells metastasized to bone (Original Magnification 400x).

Bone metastasis is a hallmark of prostate cancer dissemination, which offers an ideal setting to study the metastasis of such cancer cells within the microenvironment and specifically the molecular signals that promote their survival in the metastatic niche. To elucidate if responses to such signals in the microenvironment alters AGR-2 expression in prostate cancer cells, PC3 cells were grown in 3-dimensional spheroids and maintained for 3 days in bone marrow-conditioned medium. Total RNA was isolated from these cells and subjected to cDNA microarray analysis (**Figure 1A**), which was subsequently confirmed by real-time quantitative RT-PCR (Gene Expression Omnibus, GEO accession # GSE38714; NCBI tracking system # 16589736). AGR-2 was over-expressed significantly (p<0.05) when compared to PC3 cells cultured in regular medium (**Figure 1B**), suggesting AGR-2 might be required for initial adaptation of metastatic tumor cells to the new microenvironment. AGR-2 expression was also determined in bone metastatic prostate cancer tissue specimen. Intense AGR-2 expression was detected, which suggests requirement of AGR-2 for the establishment of bone metastasis (**Figure 1C**).

### Determination of AGR-2 mRNA Expression in Various Prostate Cancer Cell Lines

**Figure 2:**
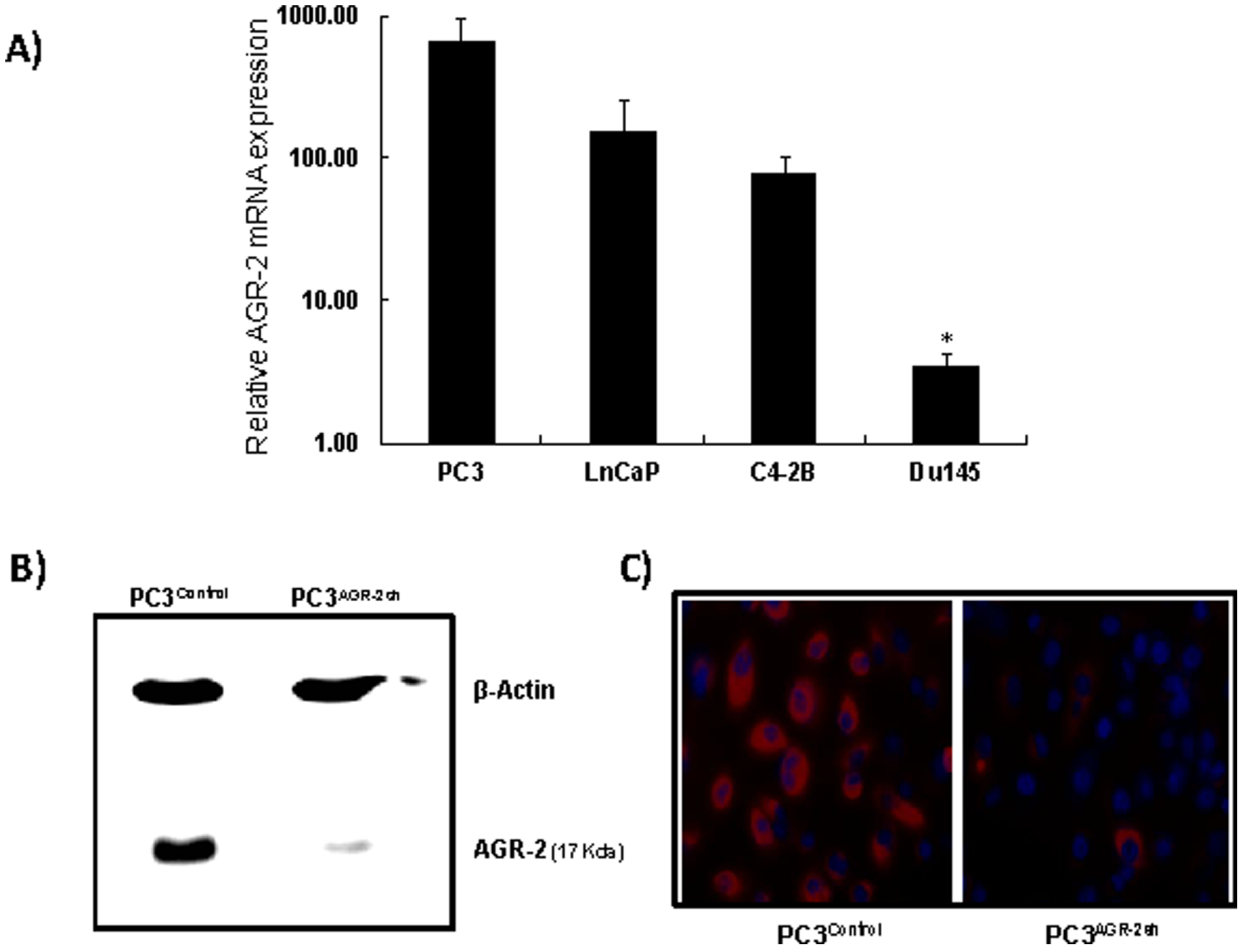
AGR-2 levels in various prostate cancer cell lines, and development and characterization of AGR-2-silenced PC3 cells. **A**. AGR-2 levels were determined by RT_PCR analysis in various human prostate cancer cell lines. The bone metastatic PC3 cells have the highest AGR-2 mRNA expression, whereas brain metastatic Du145 cells have the least amount of AGR-2 expression. **B**. **Development of AGR-2 silenced PC3 cells.** Western Blot analysis showing significant down regulation of AGR-2 protein following stable introduction of shRNA construct targeting AGR-2 in PC3 cells. beta-actin was used as a loading control. **C**. Immunofluorescence analysis comparing AGR-2 expression in AGR-2 silenced PC3 cells versus control PC3 cells (Original magnification 400X).

Relative AGR-2 mRNA expression was determined in bone metastatic PC3, LnCap and C4-2B human prostate cancer cell lines and brain metastatic Du145 prostate cancer cell line by RT-PCR. Results indicate significantly higher AGR-2 expression in PC3, LnCap and C4-2B cell lines compared to Du145 cell line, suggesting importance of AGR-2 in prostate cancer bone metastasis (**Figure 2A**).

### Determination of AGR-2 Expression in PC3^Control^ and PC3^AGR−2sh^ Cell Lines

Amount of AGR-2 gene silencing in PC3^Control^ and PC3^AGR−2sh^ cells was evaluated by Western blotting (**Figure 2B**) and immunofluorescence staining (**Figure 2C**). Over ninety percent down-regulation of AGR-2 expression was achieved in PC3^AGR−2sh^ cells when compared to PC3^Control^ cells.

### Altered Growth Characteristics of PC3 Cells Following AGR-2 Gene Silencing

**Figure 3:**
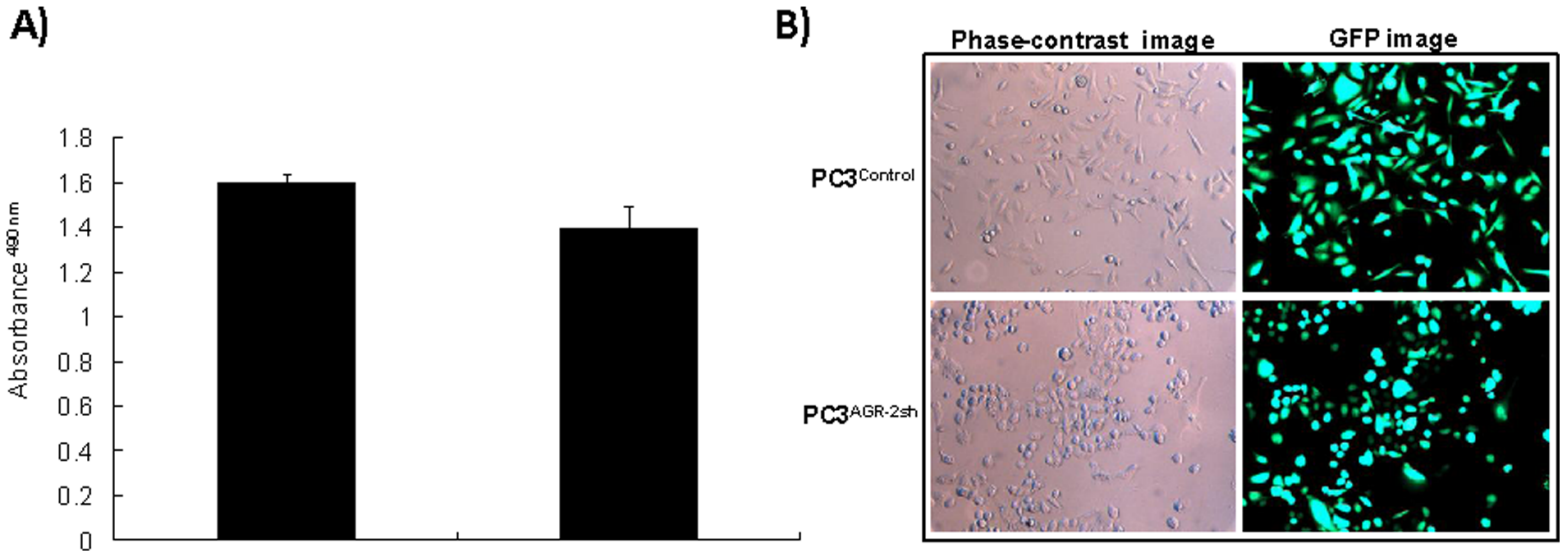
Characterization of AGR-2-silenced PC3 cells. **A**. MTT cell proliferation assay showing similar rates of growth in PC3^control^ and PC3^AGR2sh^ cells. **B**. PC3 ^control^ cells (above) showing a fibroblast-like appearance with pseudopodia-like extension for cell-cell contacts. PC3^AGR2sh^ cells (below) showed a more epithelial cell-like, rounded phenotype. These cells were also loosely attached to the culture dishes as compared to control cells. The green fluorescence in the right panels shows both PC3^control^ and PC3^AGR2sh^ cells constitutively express GFP because of the presence of GFP expressing cassette in the vectors used to create the control and AGR-2-silenced PC3 cell lines.

When PC3 cells with varying levels of AGR-2 expression were analyzed *in vitro*, there was no significant difference in proliferation rates between PC3^AGR−2sh^ and PC3^Control^ cell lines (**Figure 3A**). However, in monolayer cultures, the PC3^Control^ cells appeared to be fibroblast-like and firmly attached to the plastic surface. The cells were loosely connected to each other and only via pseudopodial extensions. The PC3^AGR−2sh^ cells on the other hand maintained more epithelial phenotype with rounded or cuboidal morphology and formed a cobblestone like appearance when confluent. Unlike the PC3^Control^ cells, PC3^AGR−2sh^ cells appeared to be loosely attached to the plastic surface (**Figure 3B**).

### AGR-2 Promotes Cellular Adhesion

**Figure 4:**
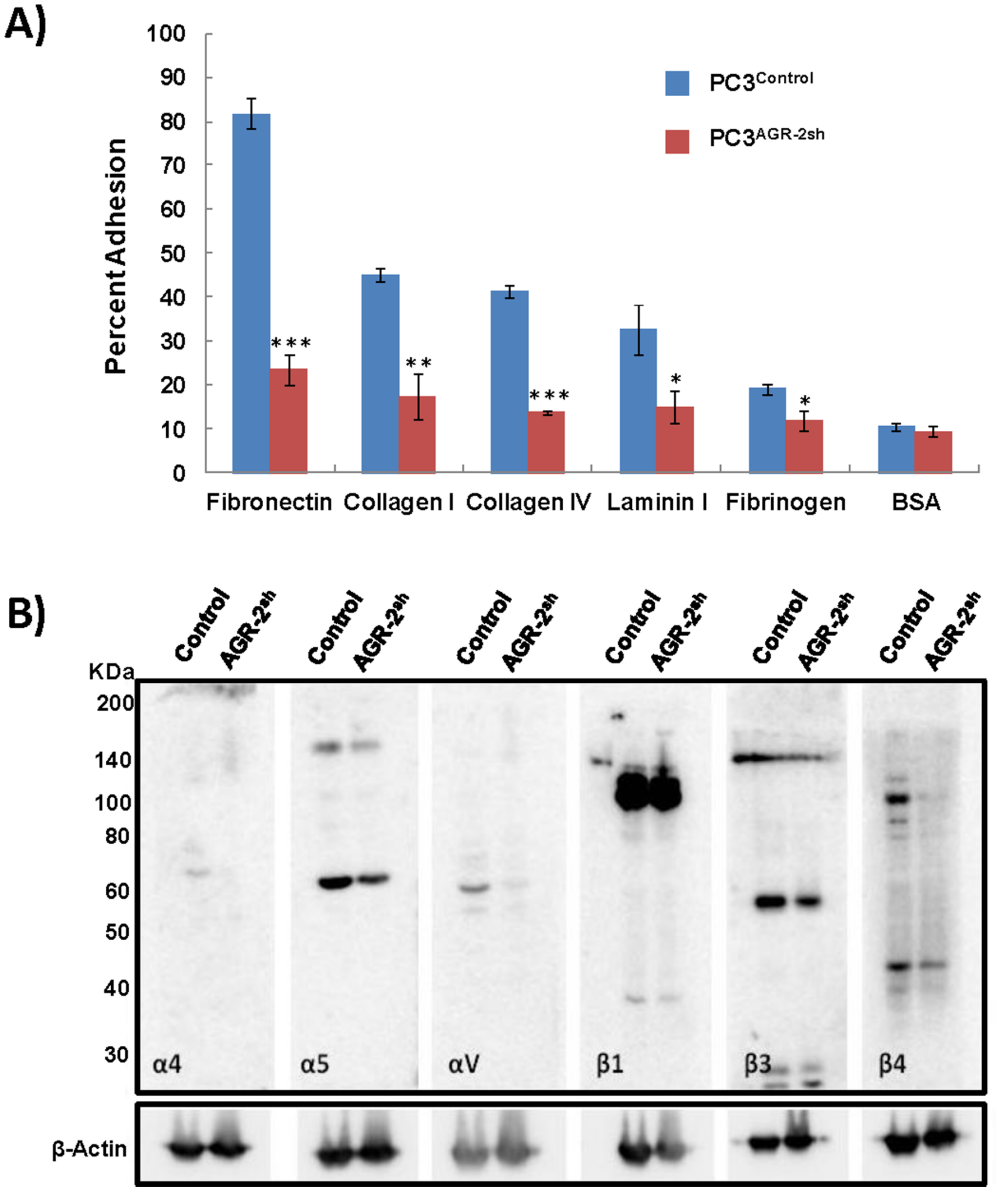
Cell adhesion properties and integrin expression of AGR-2-silenced PC3 cells. **A.** Adhesion properties of PC3^control^ and PC3^AGR2sh^ cells were evaluated following growth in culture dishes coated with various ECM proteins. First, cells were seeded in duplicate onto the coated substrates and allowed to adhere. Next, unbound cells were washed away, and the adherent cells were fixed and stained. Finally, the stain was extracted and quantified colorimetrically. Significant reduction in cellular adhesion to fibronectin (***p<0.001), collagen I (**p<0.01), collagen IV (***p<0.001), laminin (*p<0.05) and fibrinogen (*p<0.05) were observed in case of PC3^AGR2sh^ cells when compared to PC3^control^ cells. BSA coated wells were used as the reagent control (p>0.1). Data presented here are mean ± SEM. **B**. Significant down-regulation of α4, α5, αV, β3 and β4 integrins was observed in PC3^AGR2sh^ cells as compared to PC3^control^ cells. No difference in β1 integrin levels was observed between the two cell types. Beta-actin was used as the loading control. Multiple protein bands present in the blots include integrin precursor and mature proteins as well as cleaved products is expected molecular mass according to manufacturer’s information (Cell Signaling Technology, Cat# 4749S).

When PC3^Control^ and PC3^AGR−2sh^ cells were compared for their ability to bind to various extracellular matrix (ECM) proteins, including fibronectin, collagen I, collagen IV, laminin and fibrinogen utilizing a cell adhesion assay kit, results indicated significant reduction in the ability of PC3^AGR−2sh^ cells to remain attached to all the components of the ECM tested. PC3^AGR−2sh^ adhesion to fibronectin was maximally decreased (70%) among other ECM proteins, indicating AGR-2 influences pathway(s) which promote cellular adhesion (**Figure 4A**).

### Loss of Integrin Expression in PC3 Cells Lacking AGR-2

Integrin heterodimers in various combinations are known to mediate cellular adhesion to the ECM and play an important role in tumor growth and metastasis [23]. To determine if modulations of AGR-2 levels during tumor growth and metastasis is associated with altered integrin levels and function, we determined the expression of a panel of integrins (α4, α5, αV, β1, β3, β4, β5 integrins) in PC3^AGR−2sh^ and PC3^Control^ cells by Western blotting. Significantly reduced expression of α4, α5, αV, β3 and β4 integrins was observed in PC3^AGR−2sh^ cells. Comparable amounts of β1 integrin levels were observed in both cell lines whereas no β5 integrin was detected in either cell line. This data strongly suggests a role for AGR-2 in regulating cellular adhesion via integrin expression. (**Figure 4B**).

### Reduced of Tumor Cell Migration in AGR-2-silenced PC3 Cells

**Figure 5:**
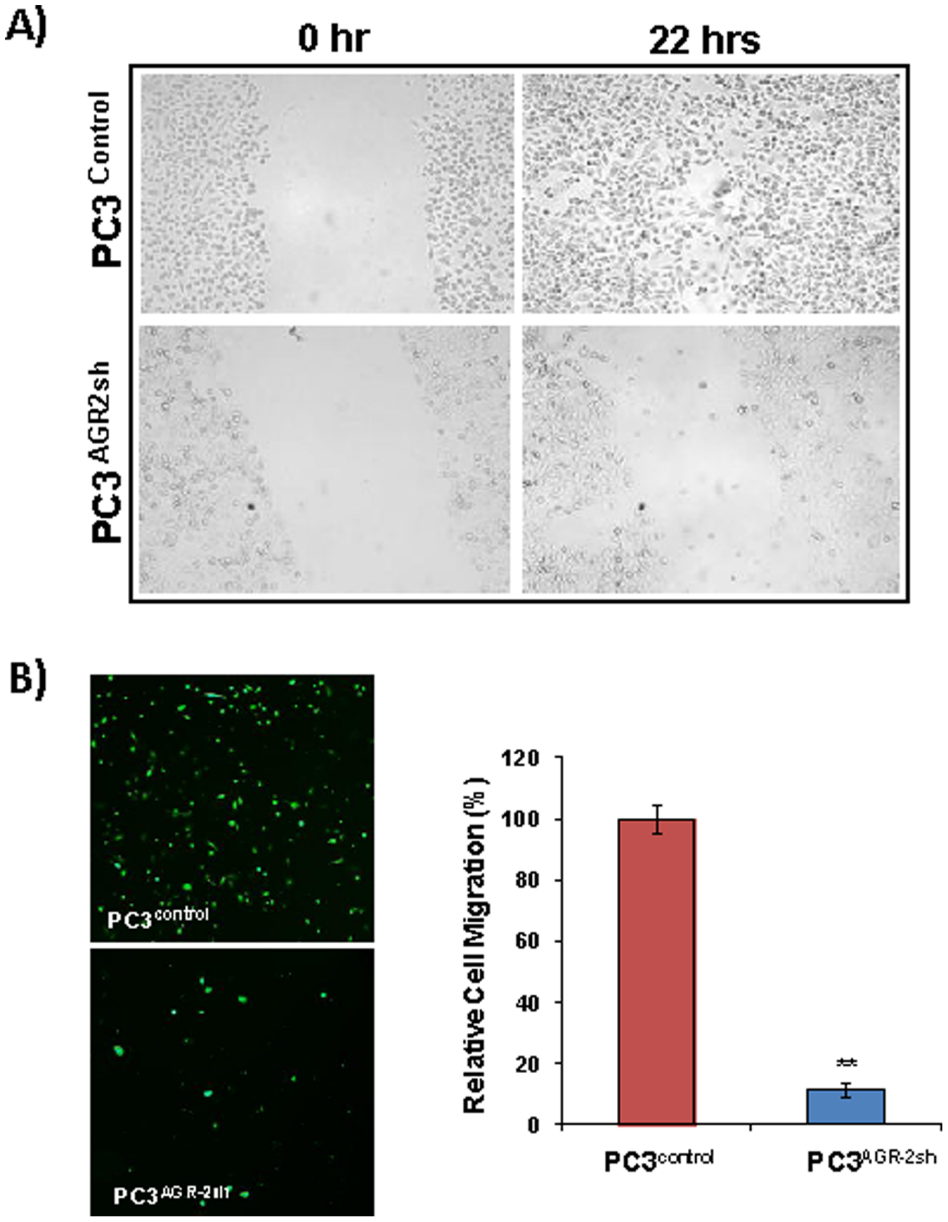
A. Wound Closure Assay. High AGR-2 expressing PC3 ^control^ cells and low AGR-2 expressing PC3 ^AGR−2sh^ cells were grown in 6 well culture dish up to 90% confluency. They were serum starved for overnight and allowed to migrate following creation of wound using a 200 µl pipette tip and adding fresh complete medium. Photographs were taken at 0 hr and 22 hrs for determination of rate of wound closure between the two cell lines. **B**. **Boyden Chamber Assay**: PC3 ^control^ cells and PC3 ^AGR−2sh^ cells were plated on cell culture inserts in serum free medium and migration of cells was stimulated adding 10% FBS as chemoattractant in the bottom chamber. After 6 hrs of incubation, cells were removed from the top of the insert using a cotton swab and cells on the underside of the insert were photographed and counted. The effect of AGR-2 silencing on PC3 cell migration is presented here as relative values compared to control (100%).

To determine if reduced cell adhesion and integrin expression in AGR-2-silenced PC3 cells affected PC3 cell migration, both a “wound closure” assay as well as a Boyden chamber migration assay was carried out. The results indicated significantly reduced (p<0.01) tumor cell migration in AGR-2-silenced PC3 cells when compared to control PC3 cells. Both these assays also suggesting cellular adhesion via integrin expression is crucial for tumor cell migration (**Figure 5** **A&B**).

### Development of TRAIL Resistance in PC3^AGR−2sh^ Cells

**Figure 6:**
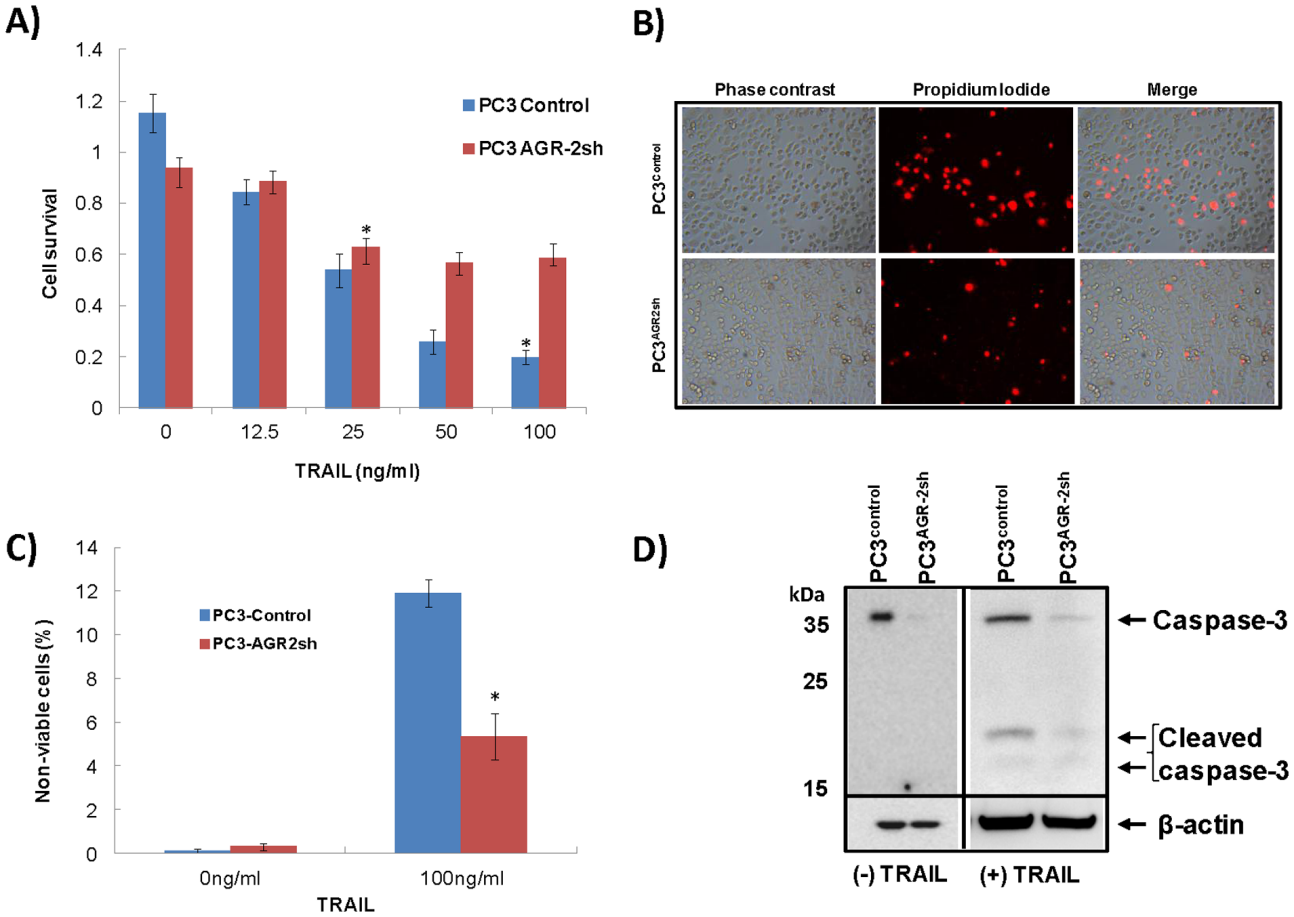
Development of TRAIL-induced death resistance in PC3 cells following AGR-2 gene silencing. **A.** PC3^control^ and PC3^AGR2sh^ cells were treated *in vitro* with various concentrations of sTRAIL. Cell viability was tested after 72 hrs using a cell viability assay kit. Significantly higher cell death (p<0.001) was observed in PC3^control^ cells compared to PC3^AGR2sh^ cells. **B**. Cell viability following sTRAIL challenge was also determined between PC3^control^ and PC3^AGR2sh^ cells by PI staining and viewing under fluorescence microscope (Original Magnification 200X). **C**. Multiple photographs were taken for each cell line following sTRAIL treatment and PI staining. Both live and dead cells were manually counted using Image J software and graphically plotted. **D**. Western blot analysis showing caspase-3 and cleaved caspase-3 levels in uninduced and TRAIL-induced control and AGR-2-silenced PC3 cells.

Previous experiments indicated AGR-2-silenced PC3 cells showed reduced cell adhesion, integrin expression and migration. Normal epithelial cells undergo apoptosis following detachment from the basement membrane (anoikis) whereas anoikis resistance is one of the hallmarks of malignant cancer cells. To determine if AGR-2 silencing has contributed to susceptibility to anoikis, both PC3^Control^ and PC3^AGR−2sh^ cells were cultured *in vitro* for 72 hrs in the presence of 0, 12.5, 25, 50 and 100 ng/ml of recombinant human soluble (s)TRAIL protein. Outcome of the experiment was analyzed by cell viability assay using a MTS cell proliferation assay kit following TRAIL treatment. Although cell death was observed in both PC3^Control^ cells and PC3^AGR−2sh^ cells, PC3^AGR−2sh^ cells survived the TRAIL challenge significantly better than the PC3^Control^ cells (**Figure 6A**) suggesting loss of AGR-2 might be associated with development of anoikis resistance in malignant tumor cells. Cell viability following sTRAIL challenge was also determined by staining the cells with propidium iodide (PI). PC3^Control^ and PC3^AGR−2sh^ cells were cultured *in vitro* for 12 hrs in the presence of 0 ng/ml and 100 ng/ml concentration of sTRAIL followed by PI staining. Phase contrast, fluorescent and overlayed images were captured using a Leica DMI 4000B microscope and analyzed with Image J software. Both live and dead cells were counted and non-viable cells (PI positive) were represented as percentage of total number of cells. Results showed significantly higher PI positive cells (p<0.05) in PC3^Control^ cells compared to PC3^AGR−2sh^ cells (**Figure 6** **B&C**). Caspase-3 is found to be activated in both extrinsic and intrinsic cell death pathways and carry out the execution phase of apoptosis [24]. Caspase-3 was found be significantly lower in AGR-2-silenced PC3 cells compared to control cells in Western blot analysis, which also supports development of anoikis resistance (**Figure 6D**). No difference was observed between control and AGR-2-silenced PC3 cells in terms of caspase-8, death receptor-5 and caspase-9 (data not shown). Caspase-3 activity requires proteolytic cleavage of inactive caspase-3 into 19/17 KDa activated cleaved caspase-3 [25]. To compare generation of cleaved caspase-3 both PC3^Control^ and PC3^AGR−2sh^ cells were grown in 50 ng/ml concentration of sTRAIL for a period of 0 hr, 1 hr, 3 hrs and 6 hrs. Cells were harvested by scrapping and cell pellet was washed with PBS twice before isolation of proteins. Caspase-3 and cleaved caspase-3 were determined by Western blotting, which showed higher levels of both peptides in PC3^Control^ cells compared to PC3^AGR−2sh^ cells in a time-dependent manner (**Figure 6D**). Analysis of prostate cancer gene expression dataset for primary and metastatic prostate cancer (194 cases) published by Taylor et al., 2010 using CBIOPORTAL indicated an odds ratio of 4.25 (confidence interval 1.40–12.86; p<0.02 by Fisher’s Exact Test) between AGR-2 and Caspase-3 suggesting a tendency towards co-occurrence of these two molecules [26].

## Discussion

Prostate cancer metastasizes to bone and produces osteoblastic/osteolytic lesions, which cause severe bone pain, susceptibility to fracture and spinal cord compression [27]. Bone metastatic cancer is incurable and leads to significant morbidity and mortality in these patients. Adhesion of exfoliated, circulating cancer cells within the bone marrow ECM proteins is the major step required for the establishment of bone metastasis [28]. In our microarray study, significant upregulation of AGR-2 mRNA expression following maintenance in bone marrow conditioned medium suggests a role of AGR-2 in facilitating the growth of prostate cancer cells in the bone microenvironment. AGR-2-silenced PC3 cells showed significantly reduced cellular adhesion to fibronectin, collagen I, collagen IV, laminin and fibrinogen, which suggests AGR-2 plays an important role in prostate cancer cell attachment within the bone marrow microenvironment. Role of AGR-2 in promoting cell adhesion is also reported in a recent study [29].

Both cell-cell and cell-ECM adhesion is mediated by integrins, which are heterodimeric transmembrane glycoproteins. αvβ3 integrin is reported to be crucial for breast and prostate cancer skeletal metastasis and binds to osteopontin, fibronectin and vitronectin [30]–[33]. The αvβ3 integrin heterodimer has been shown to be important for malignant prostate cancer cells to migrate and adhere to bone marrow matrix proteins during early stages of metastasis [34], [35]. Downregulation of both αv and β3 integrin subunits in AGR-2-silenced PC3 cells supports the earlier observations. The α5β1 integrin heterodimer has also been implicated for binding to fibronectin on human bone marrow stroma in prostate cancer [33]. Although, significant down regulation of α5 integrin was observed in our study, there was no difference in β1subunit expression between control and AGR-2-silenced PC3 cells, suggesting regulation of β1 integrins by factors other than AGR-2.

Resistance to undergo programmed cell death following detachment (anoikis) from the ECM is one of the hallmarks of epithelial cancers [36]. Kočí et al., recently showed development of resistance to TRAIL induced apoptosis in HT-29 colon cancer cells following loss of cell adhesion properties [37]. Similar observations were made in the present study in AGR-2-silenced PC3 cells, suggesting anoikis resistance in malignant cells may be associated with loss of AGR-2. In the present study, among various caspases only caspase-3 (executioner caspase) and its active fragment (cleaved caspase-3) were found to be down regulated following AGR-2 silencing in PC3 cells which explains resistance to sTRAIL mediated apoptosis in AGR-2-silenced PC3 cells and indicates that caspase-3 is a under the direct target of AGR-2. Moreover, a significantly higher odds ratio was observed between caspases-3 and AGR-2 in human prostate cancer data suggesting a strong relationship between AGR-2 and caspases-3 mediated execution steps of apoptosis. Development of resistance to extrinsic cell death pathway despite reduced tumor cell adhesion is intriguing and requires further investigation. Results of the present study clearly suggest that down-regulation of integrins, mediated by low AGR-2 levels, is initially required for the detachment of the tumor cells from the basement membrane and for the breaking of cell-cell contacts. Once they are free, these cells regain AGR-2 as well as integrin expression during their passage through the circulation and during initiation of colonies at the metastatic sites. In fact, a significantly higher AGR-2 expression was observed in circulating prostate cancer cells [38]. Loss of AGR-2 and integrin expression in higher grade tumors and enhanced AGR-2 and integrin expression in metastases support this mechanism.

Overall, AGR-2 appears to be an important molecule involved during the early stages of establishment of bone metastasis. Therefore, further studies on the role of AGR-2 in human prostate cancer and its downstream targets might lead to a better understanding of bone metastasis and may signify AGR-2 as a possible therapeutic target for preventing prostate cancer bone metastasis.

## References

[1] Jemal A, Bray F Center MM, Ferlay J, Ward E, et al (2011) Global cancer statistics. CA Cancer J Clin 61: 69–90. 21296855 10.3322/caac.20107

[2] Sturge J, Caley MP, Waxman J (2011) Bone metastasis in prostate cancer: emerging therapeutic strategies. Nat Rev Clin Oncol 8: 357–368. 21556025 10.1038/nrclinonc.2011.67

[3] Jin JK, Dayyani F, Gallick GE (2011) Steps in prostate cancer progression that lead to bone metastasis. Int J Cancer 128: 2545–2561. 21365645 10.1002/ijc.26024 PMC3082284

[4] Suva LJ, Washam C, Nicholas RW, Griffin RJ (2011) Bone metastasis: mechanisms and therapeutic opportunities. Nat Rev Endocrinol 7: 208–218. 21200394 10.1038/nrendo.2010.227 PMC3134309

[5] Kang Y, Siegel PM, Shu W, Drobnjak M, Kakonen SM, et al (2003) A multigenic program mediating breast cancer metastasis to bone. Cancer Cell 3: 537–549. 12842083 10.1016/s1535-6108(03)00132-6

[6] Aberger F, Weidinger G, Grunz H, Richter K (1998) Anterior specification of embryonic ectoderm: the role of the Xenopus cement gland-specific gene XAG-2. Mech Dev 72: 115–130. 9533957 10.1016/s0925-4773(98)00021-5

[7] Park SW, Zhen G, Verhaeghe C, Nakagami Y, Nguyenvu LT, et al (2009) The protein disulfide isomerase AGR2 is essential for production of intestinal mucus. Proc Natl Acad Sci U S A 106: 6950–6955. 19359471 10.1073/pnas.0808722106 PMC2678445

[8] Pizzi M, Fassan M, Balistreri M, Galligioni A, Rea F, et al (2012) Anterior gradient 2 overexpression in lung adenocarcinoma. Appl Immunohistochem Mol Morphol 20: 31–36. 21768879 10.1097/PAI.0b013e3182233f9f

[9] Marin-Aguilera M, Mengual L, Ribal MJ, Ars E, Rios J, et al. (2012) Utility of urothelial mRNA markers in blood for staging and monitoring bladder cancer. Urology 79: 240 e249–215. 10.1016/j.urology.2011.09.006 22055693

[10] Lee HJ, Hong CY, Kim MH, Lee YK, Nguyen-Pham TN, et al (2012) In vitro induction of anterior gradient-2-specific cytotoxic T lymphocytes by dendritic cells transduced with recombinant adenoviruses as a potential therapy for colorectal cancer. Exp Mol Med 44: 60–67. 22089087 10.3858/emm.2012.44.1.006 PMC3277899

[11] Lee do H, Lee Y, Ryu J, Park SG, Cho S, et al (2011) Identification of proteins differentially expressed in gastric cancer cells with high metastatic potential for invasion to lymph nodes. Mol Cells 31: 563–571. 21533548 10.1007/s10059-011-1053-z PMC3887625

[12] Nancarrow DJ, Clouston AD, Smithers BM, Gotley DC, Drew PA, et al (2011) Whole genome expression array profiling highlights differences in mucosal defense genes in Barrett’s esophagus and esophageal adenocarcinoma. PLoS One 6: e22513. 21829465 10.1371/journal.pone.0022513 PMC3145652

[13] Maresh EL, Mah V, Alavi M, Horvath S, Bagryanova L, et al (2010) Differential expression of anterior gradient gene AGR2 in prostate cancer. BMC Cancer 10: 680. 21144054 10.1186/1471-2407-10-680 PMC3009682

[14] Wang Z, Hao Y, Lowe AW (2008) The adenocarcinoma-associated antigen, AGR2, promotes tumor growth, cell migration, and cellular transformation. Cancer Res 68: 492–497. 18199544 10.1158/0008-5472.CAN-07-2930

[15] Liu D, Rudland PS, Sibson DR, Platt-Higgins A, Barraclough R (2005) Human homologue of cement gland protein, a novel metastasis inducer associated with breast carcinomas. Cancer Res 65: 3796–3805. 15867376 10.1158/0008-5472.CAN-04-3823

[16] Barraclough DL, Platt-Higgins A, de Silva Rudland S, Barraclough R, Winstanley J, et al (2009) The metastasis-associated anterior gradient 2 protein is correlated with poor survival of breast cancer patients. Am J Pathol 175: 1848–1857. 19834055 10.2353/ajpath.2009.090246 PMC2774050

[17] Zhang Y, Forootan SS, Liu D, Barraclough R, Foster CS, et al (2007) Increased expression of anterior gradient-2 is significantly associated with poor survival of prostate cancer patients. Prostate Cancer Prostatic Dis 10: 293–300. 17457305 10.1038/sj.pcan.4500960

[18] Dong A, Gupta A, Pai RK, Tun M, Lowe AW (2011) The human adenocarcinoma-associated gene, AGR2, induces expression of amphiregulin through Hippo pathway co-activator YAP1 activation. J Biol Chem 286: 18301–18310. 21454516 10.1074/jbc.M110.215707 PMC3093902

[19] Dumartin L, Whiteman HJ, Weeks ME, Hariharan D, Dmitrovic B, et al (2011) AGR2 is a novel surface antigen that promotes the dissemination of pancreatic cancer cells through regulation of cathepsins B and D. Cancer Res. 71: 7091–7102. 10.1158/0008-5472.CAN-11-1367 PMC3541941 21948970

[20] Maslon MM, Hrstka R, Vojtesek B, Hupp TR (2010) A divergent substrate-binding loop within the pro-oncogenic protein anterior gradient-2 forms a docking site for Reptin. J Mol Biol 404: 418–438. 20888340 10.1016/j.jmb.2010.09.035

[21] Zhang JS, Gong A, Cheville JC, Smith DI, Young CY (2005) AGR2, an androgen-inducible secretory protein overexpressed in prostate cancer. Genes Chromosomes Cancer 43: 249–259. 15834940 10.1002/gcc.20188

[22] Tuschl T, Zamore PD, Lehmann R, Bartel DP, Sharp PA (1999) Targeted mRNA degradation by double-stranded RNA in vitro. Genes Dev 13: 3191–3197. 10617568 10.1101/gad.13.24.3191 PMC317199

[23] Rathinam R, Alahari SK (2010) Important role of integrins in the cancer biology. Cancer Metastasis Rev 29: 223–237. 20112053 10.1007/s10555-010-9211-x

[24] Chowdhury I, Tharakan B, Bhat GK (2008) Caspases - an update. Comp Biochem Physiol B Biochem Mol Biol 151: 10–27. 18602321 10.1016/j.cbpb.2008.05.010

[25] Nicholson DW, Ali A, Thornberry NA, Vaillancourt JP, Ding CK, et al (1995) Identification and inhibition of the ICE/CED-3 protease necessary for mammalian apoptosis. Nature 376: 37–43. 7596430 10.1038/376037a0

[26] Taylor BS, Schultz N, Hieronymus H, Gopalan A, Xiao Y, et al (2010) Integrative genomic profiling of human prostate cancer. Cancer Cell 18: 11–22. 20579941 10.1016/j.ccr.2010.05.026 PMC3198787

[27] Roudier MP, Morrissey C, True LD, Higano CS, Vessella RL, et al (2008) Histopathological assessment of prostate cancer bone osteoblastic metastases. J Urol 180: 1154–1160. 18639279 10.1016/j.juro.2008.04.140 PMC2992811

[28] Schneider JG, Amend SR, Weilbaecher KN (2011) Integrins and bone metastasis: integrating tumor cell and stromal cell interactions. Bone 48: 54–65. 20850578 10.1016/j.bone.2010.09.016 PMC3010439

[29] Patel P, Clarke C, Barraclough DL, Jowitt TA, Rudland PS, et al (2013) Metastasis-promoting anterior gradient 2 protein has a dimeric thioredoxin fold structure and a role in cell adhesion. J Mol Biol 425: 929–943. 23274113 10.1016/j.jmb.2012.12.009

[30] van der P, Vloedgraven H, Papapoulos S, Lowick C, Grzesik W, et al (1997) Attachment characteristics and involvement of integrins in adhesion of breast cancer cell lines to extracellular bone matrix components. Lab Invest 77: 665–675. 9426405

[31] Liapis H, Flath A, Kitazawa S (1996) Integrin alpha V beta 3 expression by bone-residing breast cancer metastases. Diagn Mol Pathol 5: 127–135. 8727100 10.1097/00019606-199606000-00008

[32] McCabe NP, De S, Vasanji A, Brainard J, Byzova TV (2007) Prostate cancer specific integrin alphavbeta3 modulates bone metastatic growth and tissue remodeling. Oncogene 26: 6238–6243. 17369840 10.1038/sj.onc.1210429 PMC2753215

[33] Van der Velde-Zimmermann D, Verdaasdonk MA, Rademakers LH, De Weger RA, Van den Tweel JG, et al (1997) Fibronectin distribution in human bone marrow stroma: matrix assembly and tumor cell adhesion via alpha5 beta1 integrin. Exp Cell Res 230: 111–120. 9013713 10.1006/excr.1996.3405

[34] Nakamura I, Duong le T, Rodan SB, Rodan GA (2007) Involvement of alpha(v)beta3 integrins in osteoclast function. J Bone Miner Metab 25: 337–344. 17968485 10.1007/s00774-007-0773-9

[35] Barthel SR, Hays DL, Yazawa EM, Opperman M, Walley KC, et al (2013) Definition of molecular determinants of prostate cancer cell bone extravasation. Cancer Res 73: 942–952. 23149920 10.1158/0008-5472.CAN-12-3264 PMC3548951

[36] Paoli P, Giannoni E, Chiarugi P (2013) Anoikis molecular pathways and its role in cancer progression. Biochim Biophys Acta. 10.1016/j.bbamcr.2013.06.026 23830918

[37] Koci L, Hyzd’alova M, Vaculova A, Hofmanova J, Kozubik A (2011) Detachment-mediated resistance to TRAIL-induced apoptosis is associated with stimulation of the PI3K/Akt pathway in fetal and adenocarcinoma epithelial colon cells. Cytokine 55: 34–39. 21482133 10.1016/j.cyto.2011.03.013

[38] Smirnov DA, Zweitzig DR, Foulk BW, Miller MC, Doyle GV, et al (2005) Global gene expression profiling of circulating tumor cells. Cancer Res 65: 4993–4997. 15958538 10.1158/0008-5472.CAN-04-4330

